# Ultra-sensitive micro thermoelectric device for energy harvesting and ultra-low airflow detection

**DOI:** 10.1038/s41378-025-00921-7

**Published:** 2025-04-28

**Authors:** Bo Yan, Jiaxiang Wang, Yi Chen, Yahui Li, Xiangxiang Gao, Zhiyuan Hu, Xiaowen Zhou, Mengqiu Li, Zhuoqing Yang, Congchun Zhang

**Affiliations:** 1 National Key Laboratory of Advanced Micro and Nano Manufacture Technology, Shanghai Jiao Tong University, Shanghai, 200240 China; 2 Department of Micro/Nano Electronics, School of Electronic Information and Electrical Engineering, Shanghai Jiao Tong University, Shanghai, 200240 China; 3 School of Electrical and Electronic Engineering, Nanyang Technological University, Singapore, 639798 Singapore

**Keywords:** Sensors, Electrical and electronic engineering

## Abstract

Micro thermoelectric device (μ-TED) emerges with great attention in energy generation, thermal management, and heat sensing applications. However, the large sensitive area is necessary to accommodate enough thermoelectric couples (TCs) for a high thermoelectric performance. This limits the potential in micro energy harvesting and ultra-sensitive sensing applications. Here, we adopted an optimized MEMS-based process to fabricate the ultra-sensitive micro-thermoelectric device (μ-TED). With the help of MEMS-compatible electrochemical deposition, the small size (25 μm), high aspect ratio (1:1.25), and alternating distributed P/N structures are achieved. As a result, the μ-TED realizes an ultra-high integration density of 19,900 thermoelectric couples per cm^2^. Moreover, it shows a great thermoelectric sensitivity of 212 mV/(K·cm^2^) and a competitive power factor of 0.51 μW/(K^2^·cm^2^), which means the μ-TED is competent for miniaturized applications. Additionally, the μ-TED shows an ultra-low detection limit of 5 mm/s and a short response time of 100 ms, revealing great potential in fast detections of the ultra-low airflow. Furthermore, the ultra-sensitive μ-TED is utilized as a flexible breath sensor, due to its compact size. The breath signal of different motion states is successfully detected. These results confirm that the ultra-sensitive μ-TED holds outstanding potential for ultra-sensitive airflow sensing and energy harvesting devices.

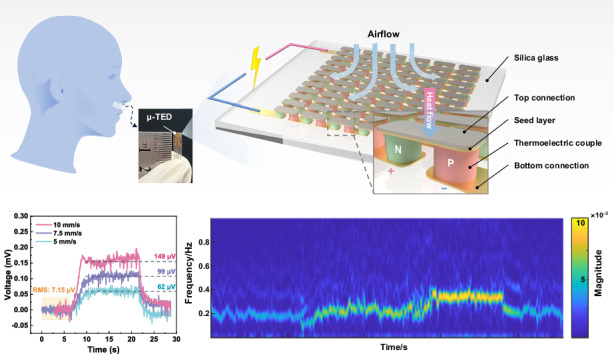

## Introduction

Airflow sensors have been witnessed to undergo explosive development in lots of fields, such as climatic, aerospace, and human health monitoring^1–6^. Recently, much attention has been focused on ultra-sensitive and fast-response airflow detections. For high-sensitivity applications, thin-film resistance sensors have realized airflow detection below 100 mm/s^7^. At the same time, piezoresistive airflow sensors explored many advanced materials to improve the detection performance, such as graphene and carbon nanotubes (CNTs)^2–4,8,9^. A highly sensitive airflow sensor based on in situ-grown CNTs was reported with a detection limit of 50 mm/s^2^. Graphene/CNT-based airflow sensors further achieved the detection of 17.6 mm/s airflow^3^. However, the high sensitivity is at the cost of a large sensitive area, which is adverse to response speed. The response time mostly ranges from 0.5 to 1 s^2–4,8^, hard to satisfy the demand for real-time monitoring. The fiber-optic airflow sensors are superior at fast detection. An airflow sensor based on micro/nanofiber has been reported with an ultra-fast response time of 12 ms^10^. Unfortunately, the complex instruments limit the applications, including expensive light sources and spectrometers. Up to now, the reported airflow sensors are hard to satisfying both requirements of ultra-low detection limit and fast response time. Urgent efforts are required to promote the common improvement in detection limit and response speed.

Thermoelectric device (TED) is a superior candidate for high-sensitive detections because of its excellent energy generation and heat sensing ability^11–20^. Typically, the sensitivity of TED can be simply improved by increasing the amounts of thermocouples (TCs). Therefore, MEMS-based (micro-electro-mechanical system) micro thermoelectric device (μ-TED)^21–23^ exhibits a promising potential due to the high-integration ability. The μ-TED of 10082 TCs (in 4 cm^2^ area) is reported to successfully detect the airflow of 4 mm/s^21^ with a response time of 1.7 s. A large sensitive area is needed for high sensitivity, which is harmful to the response speed. The size of TCs remains to be reducing, and quick-response μ-TED is in urgent need of further research.

For fast detection, low-dimension structures featuring ultra-small volume are demonstrated of positive improvements^1,9,24,25^. Recently reported airflow sensors with suspended CNT networks show an ultra-fast response (~21 ms)^9^. Single silicon nanowires also exhibit outstanding transient response performance, owning a response time of 40 ms^24^. Moreover, our previous work on light-driven μ-TED has achieved a fast light response (4.2 ms) by miniaturizing the light absorb layer^22^. Fast response (8 ms) to IR laser is also demonstrated on μ-TED^5^. The top connections of the μ-TED play a similar role in airflow detection. Hence, the response speed of the μ-TED is expected to be improved by miniaturizing the top connections. Further miniaturization of the μ-TED is beneficial to the both improvement of sensitivity and response speed. High-integration μ-TED is of great significance in highly sensitive and fast response airflow detections.

In this work, we report the fabrication of an ultra-sensitive micro-thermoelectric device (μ-TED) utilizing an optimized MEMS-based process. The high-performance Bi_2_Te_3_/Sb_2_Te_3_ thermoelectric structures are remarkably reduced to 25 μm via electrochemical deposition (ECD). Technical difficulties in high aspect ratio (1:1.25) and alternating distributed P/N structures are overcome. As a result, this work achieved an extremely high integration density of 19,900 TCs/cm^2^. Additionally, the suspended top connections were miniaturized alongside the thermoelectric structures, enhancing the transient response performance. Thereafter, the thermoelectric performance and power generation ability were thoroughly investigated. Also, the airflow sensing mechanism of μ-TED is discussed, and the sensing performance of ultra-low airflow is examined. Moreover, the ultra-sensitive μ-TED was employed as a flexible breath sensor to demonstrate its potential for miniaturized and portable applications.

## Results and discussion

### Design concept of the ultra-sensitive μ-TED

**Fig. 1:**
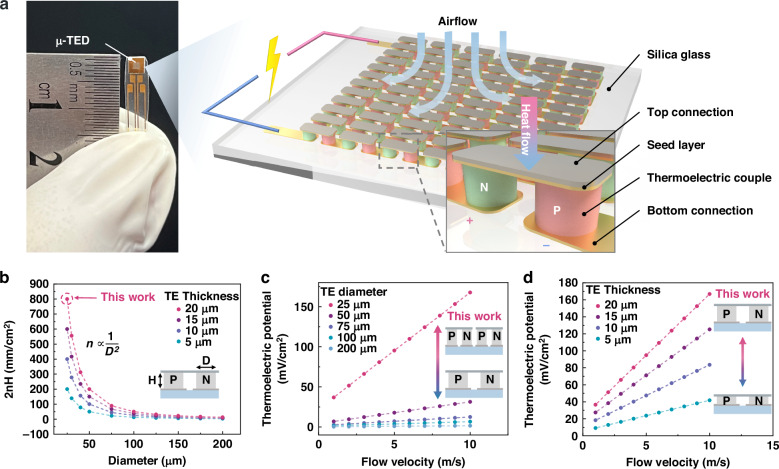
Schematic illustration of the ultra-sensitive μ-TED for airflow detection. **a** The practicality picture and 3D visualization of the μ-TED. **b** The 2nH changes with diameter D and thickness H of TE elements. Here, 2nH represents the total length of the thermoelectric legs per unit area. **c** Airflow sensing simulation of μ-TED with different TE element diameters. **d** Airflow sensing simulation of μ-TED with different TE element thickness

Figure 1 shows the ultra-sensitive micro thermoelectric device (μ-TED) and the schematic diagram of the airflow sensing application. The μ-TED consists of P and N-type thermoelectric elements with opposite Seebeck coefficients. The minimum units, thermoelectric couples (TCs), each contain a pair of P/N elements and connect in series. Based on the Seebeck effect, the μ-TED could convert temperature difference into electrical signal in a reversible and all-solid-state way^11^:

1 $$U=n{S}_{{PN}}\Delta T$$

where n is the amounts of thermoelectric couples (TCs) per unit area, and S_PN_ is the Seebeck coefficient of the P/N thermoelectric couple, ΔT is the temperature difference along thermoelectric units. The temperature difference is closely related to the heat transfer process, which enables the μ-TED to naturally own the ability of heat sensing and power generation. When the air flows through the μ-TED surface, the air heat convection Q_air_ is generated perpendicular to the device:

2 $${Q}_{{air}}={h}_{v}{A}_{T}{(T}_{{air}}-{T}_{\mu -{TED}})$$

where *h*_*ν*_ is the convective heat transfer coefficient, A_T_ is approximated to be the area of the top connection of the μ-TED, and T_air_ and T_μ-TED_ are the temperature of the airflow and the μ-TED, respectively. Among that, the convective heat transfer coefficient h_ν_ is highly dependent on the flow velocity in reference^26^:

3 $${h}_{v}=a+{bv}$$

where a and b are the empirical coefficients in practical experiments, and *ν* is the airflow velocity.

According to Fourier’s heat transfer theory, the heat flow Q_TE_ flowing through the thermoelectric element can be described as the following formula:

4 $${Q}_{{TE}}={Q}_{{air}}=-\lambda {A}_{{TE}}\frac{\Delta T}{H}$$

where λ is the thermal conductivity of thermoelectric material, A_TE_ is the cross-section area of the thermoelectric elements, and ΔT and H are the temperature difference and thickness of the thermoelectric elements, respectively.

According to formula (1)–(4), the thermoelectric potential U can be expressed as a function of airflow velocity *ν*:

5 $$U(v,{T}_{\mu -{TED}})=-{nH}\frac{{A}_{T}{S}_{{PN}}}{{A}_{{TE}}\lambda }(a+{bv})({T}_{{air}}-{T}_{\mu -{TED}})$$

Formula (5) clearly shows the airflow sensing mechanism of the μ-TED and its influence factor. The structural features contribute a lot to sensing performance, such as the top connection area A_T_, the sectional area A_TE_, the TC density n, and the TE elements thickness H.

Among them, the A_T_ and A_TE_ are linearly dependent, and A_T_/A_TE_ can be approximated to a constant c (around 3.03 in this work). Formula (5) can be simplified as follows:

6 $$U(v,{T}_{\mu -{TED}})=-2{nH}\frac{{S}_{{PN}}}{\lambda }\cdot \frac{c}{2}(a+{bv})({T}_{{air}}-{T}_{\mu -{TED}})$$

where the 2nH stands for the total length of the thermocouples (TCs) legs per unit area, which effectively contributes to the thermoelectric performance. Formula (6) indicates the total length of the TCs is the key factor of the airflow sensing performance.

Further, numerical analysis of the μ-TED airflow sensing performance is performed (see the Methods section). Here, the room temperature is set as 20 °C, and the bottom temperature of the μ-TED device is fixed at a constant temperature of 30 °C. For the structural parameters see Supplementary Note S1 and Supplementary Fig. S1a, Supplementary Information. As shown in simulation result Fig. 1b, the decrease of the TE element diameter D exponentially contributes to the increase of the 2nH, which will greatly enhance the airflow sensing signal U(*v*, T_μ-TED_). The influence of the TE element thickness H is relatively gentle, close to a linear relationship. The airflow detection simulations are performed as shown in Fig. 1c, d. They confirm the great influence of 2nH on the airflow sensing performance. Hence, the high-integration miniaturized μ-TED is of great importance in ultra-sensitive airflow sensing.

Actually, the ultra-sensitive μ-TED is based on the highly integrated TCs. Here, we fabricated an ultra-high integration μ-TED with the MEMS-based process. High-performance thermoelectric materials, N-type Bi_2_Te_3_ and P-type Sb_2_Te_3_, are introduced as TE elements due to their outstanding thermoelectric performance. Particularly, their size is reduced to 25 μm diameter, and the integration density is high to 19,900 TCs/cm^2^. Besides, the thick TE elements of 20 μm are achieved with a MEMS-compatible electrochemical deposition (ECD). These features make the as-reported μ-TED very competitive in power generation and airflow sensing applications.

### Fabrication process of the ultra-sensitive μ-TED

**Fig. 2:**
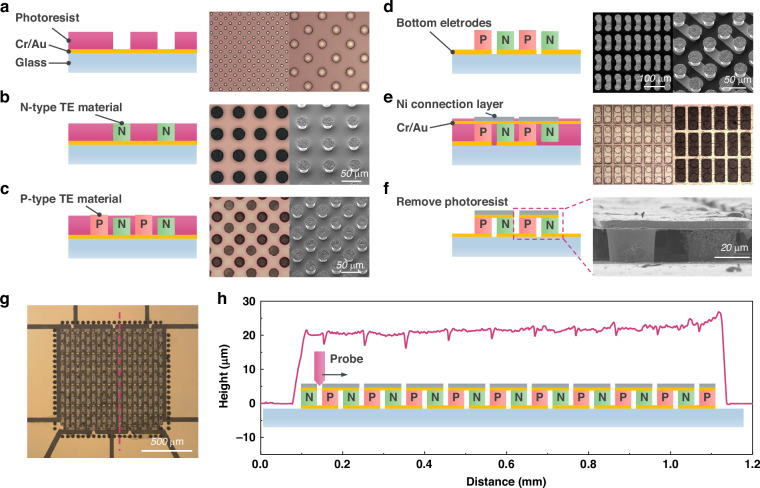
Fabrication and micro-morphology of the ultra-sensitive μ-TED. **a**, **f** The MEMS-based fabrication process of the μ-TED. **a** Fabrication of bottom seed layer and photolithograph of n-type pattern. **b** Electrochemical deposition of n-type Bi_2_Te_3_. **c** Photolithograph and electrochemical deposition of p-type Sb_2_Te_3_. **d** Photolithographing etching of bottom electrode pattern. **e** Electroplate of top connection layer. **f** Etching top seed layer and releasing residual photoresist. **g** Optical image of as-fabricated μ-TED. The red line means the test region of the step profiler. **h** The height data of the top surface of the μ-TED

The MEMS-based fabrication process of the ultra-sensitive μ-TED is described in Fig. 2a–f. Firstly, an Au seed layer of 1-μm thickness is sputtered on the silica glass substrate with a Cr adhesion layer of 30 nm. Here, the Au is chosen as the bottom layer because of its good resistance to nitric acid. A thick photoresist of 20 μm thickness is spin-coated on the substrate, and the N-type pattern is developed by the photolithography process (Fig. 2a). Secondly, the N-type Bi_2_Te_3_ is fabricated directly on the N-type pattern by an electrochemical deposition (ECD) method (Fig. 2b). Next, a thin photoresist (2 μm) is spin-coated, and P-type Sb_2_Te_3_ is deposited by ECD (Fig. 2c). Then, the bottom seed layer is etched to form the bottom electrodes (Fig. 2d). In the steps of Fig. 2c, d, the sample must strictly avoid to be exposed to UV-light. After that, a thick photoresist is coated and polished to the same height with thermoelectric elements. The top seed layer Cr/Au is sputtered and a Ni connection layer (2 um) is fabricated by photolithography and electroplating (Fig. 2e). The ductility of Au is excellent which could withstand the subsequent processes above the thick photoresist. Finally, the useless part of the top seed layer is etched, and the residual photoresist is removed by acetone (Fig. 2f). The detailed structure image during each process is shown in Supplementary Fig. S2, Supplementary information.

Figure 2g displays the μ-TED image of the optical microscope. The sensitive area is defined as 1 mm*1 mm, which contains 199 thermoelectric couples (TCs). The integration density reaches 19,900 TCs/cm^2^. Figure 2h shows the height information of the red line in Fig. 2g. The TE elements are not distinguished from each other, because the probe owns a large angle and can’t get down to the bottom between the TE elements. The overall height of the μ-TED is about 22 μm.

Besides, Bi_2_Te_3_ and Sb_2_Te_3_ samples without photolithography patterns are prepared with the same ECD conditions. XRD and EDS tests are performed to investigate the phase composition, as shown in Supplementary Fig. S3 (Supplementary information). Results indicate that Bi_2_Te_3_ and Sb_2_Te_3_ are successfully fabricated with the ECD condition in Experimental Section “Fabrication details of the ultra-sensitive μ-TED”. Further, the element composition of the μ-TED is studied by the EDS test, as shown in Supplementary Fig. S4 (Supplementary information). The Bi_2_Te_3_ is successfully prepared with Bi:Te ≈ 2:3. In the Sb_2_Te_3_ area, the ratio of Sb:Te is 2:4.56. There is no other possible chemical compound composed of Sb and Te. Hence, the P-type TE material may be composed of 76% Sb_2_Te_3_ and 24% Te. We think it is due to the slow diffusion velocity of Sb^3+^, which is indirectly dissolved in the ECD solution through a tartaric (organic) acid. In the micro-size opening, the diffusion of Sb^3+^ is not efficient for the Sb_2_Te_3_ deposition. Hence, additional adjustment is required for the ECD deposition of micro-size Sb_2_Te_3_ structure in future research.

### Thermoelectric performance of the ultra-sensitive μ-TED

**Fig. 3:**
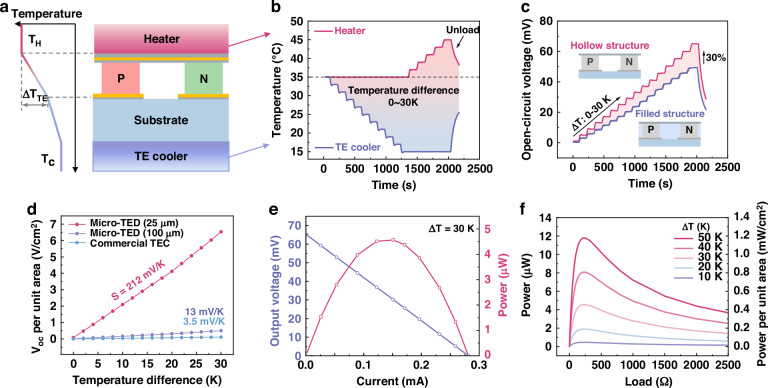
Thermoelectric performance of the ultra-sensitive μ-TED. **a** Temperature difference platform for thermoelectric performance test. **b** The temperature curve of the hot and cool sides of the temperature difference platform. **c** The thermoelectric performance comparison of the μ-TED before/after removing the photoresist at 0–30 K temperature difference. The red line means a hollow structure (without photoresist), and the blue line means a filled structure (filled with the photoresist). **d** Voltage density of different structures TED. **e** The output performance of μ-TED with different external resistance loads. **f** The power generation ability of μ-TED with matched external resistance under different temperature differences

The micro thermoelectric device (μ-TED) converts temperature difference into electric energy with the Seebeck effect. Thermoelectric performance is of great importance in power generation and sensing applications. Here, a homemade dual temperature control platform is introduced to provide ΔT perpendicular to the μ-TED, as shown in Fig. 3a (The platform is shown in Supplementary Fig. S5, Supplementary information). The temperature of the top and bottom of the platform is controlled with a resistance heater and a thermoelectric (TE) cooler, respectively. The open-circuit voltage of the μ-TED is continuously measured by a voltmeter. The environmental humidity of the testing is in the range of 40–45% RH. For instance, a step-increasing temperature difference is applied in Fig. 3b, and the corresponding open-circuit voltage is recorded in Fig. 3c. Here, the hollow structure and the filled structure are compared, which represent μ-TEDs before and after releasing photoresist. A 30% improvement in thermoelectric voltage is observed after releasing the photoresist. This is due to that the average thermal resistance increases and the larger temperature difference concentrates on the TE elements. To illustrate the advantage of the as-reported μ-TEDs, the output voltage density is compared with two types of thermoelectric devices of different TE element diameters. Here, the reference devices are a 4 mm*4 mm micro-TED (280 TCs; diameter: 100 μm) and a 20 mm* 20 mm commercial TEC (49 TCs; square: 1.4*1.4 mm). The as-reported μ-TED shows a thermoelectric sensitivity of 212 mV/(K·cm^2^), which is over 15 times better than the others. This indicates the superiority of the ultra-sensitive μ-TED. The detailed comparison with other recent research will be discussed later.

Figure 3e shows the current and the output voltage with different external resistance at ΔT = 30 K. Along with the increase of external resistance, output power increases first and then decreases, reaching a maximum value of 4.5 μW at R_external_=R_internal_. For this work, the average resistance of the μ-TED is about 232 Ω. Typically, the output power directly relies on the temperature difference distributed on the μ-TED. The thermoelectric performance of temperature difference of 10–50 K is supplemented in Supplementary Fig. S5, Supplementary information. And, the energy outputs performance of them are shown in Fig. 3f. The as-reported μ-TED could generate an output power of 1.18 mW/cm^2^ at ΔT = 50 K, showing a good power generation ability.

### Ultra-low airflow sensing performance of the ultra-sensitive μ-TED

**Fig. 4:**
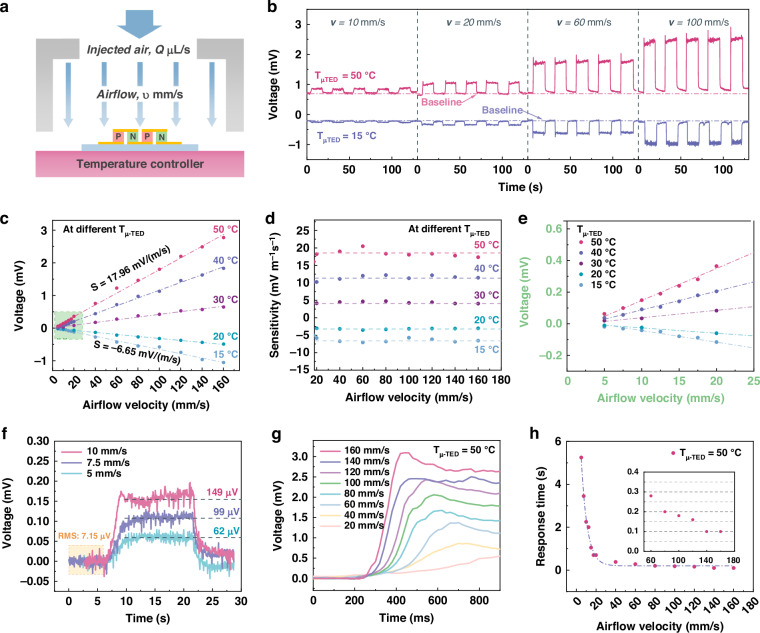
Airflow sensing performance of the ultra-sensitive μ-TED. **a** The schematic diagram of the airflow measurement. **b** The open-circuit voltage of the μ-TED under different flow velocities and device temperature. **c** Voltage signal of 5–160 mm/s under different device temperatures after removing the baseline. **d** The sensitivity of different airflow velocities under the μ-TED temperature of 15–50 °C. **e** Detailed voltage signal of the μ-TED at 5–20 mm/s under different device temperatures. **f** The voltage signal of ultra-low airflow at a device temperature of 50 °C. **g** The transient response of the μ-TED for different airflow velocities. **h** The response time of the μ-TED changes with the airflow velocity

The airflow sensing experiment is performed to study the sensing performance of the μ-TED. A schematic diagram of the ultra-low airflow detection is shown in Fig. 4a. The air is injected with a homemade motoring syringe at a certain rate Q μL/s, which converts into an airflow of velocity *v* mm/s according to the outlet diameter. The relationship between the injected air rate and the airflow velocity *v* see Supplementary Table S1.2 of Supplementary Note S1, Supplementary information. During the experiment, the μ-TED is placed on a temperature controller to maintain the device temperature (T_μ-TED_), and the injected air is the same as room temperature (T_air_ = 23 °C). And, the environmental humidity is in the range of 40–45% RH. Figure 4b shows the open-circuit voltage of the μ-TED under different flow velocities and device temperature. The voltage signal increases gradually along with the increase of the airflow velocity. The voltage signal of the 50 °C device is higher than that of the 15 °C device, and they are going in the opposite direction. This is due to the direction of the temperature difference (T_air_ - T_μ-TED_). The baseline under no airflow is not zero. This is in agreement with the formulas (3) and (6), as the constant *a* is not equal to zero. Even the external flow velocity is down to zero, the temperature difference between the μ-TED device and the air leads the surrounding air to flow spontaneously, which is namely the natural thermal convection.

To clearly study the relationship of thermoelectric voltage vs flow velocity *v*, the baseline is recorrected to zero. The corresponding voltage signal is shown in Fig. 4c, where the airflow velocity ranges from 5 to 160 mm/s. The thermoelectric voltage U shows a linear relation with the flow velocity *v* under each device temperature. This is due to the linear change of the heat transfer coefficient *h*, following the formula (3) and (6). The sensitivity, defined as U/*v* for each airflow velocity, is calculated in Fig. 4d. It shows good stability among airflow velocity of 20–160 mm/s. The sensitivity values from 15 to 50 °C are −6.65, −3.08, 4.16, 11.91, and 17.96 mV/(m/s), respectively. Moreover, the as-reported μ-TED could detect ultra-low airflow below 20 mm/s, as shown in Fig. 4e. It still shows a good linearity at a flow velocity of 5–20 mm/s. The corresponding sensitivity is shown in Supplementary Fig. S6d, Supplementary information. The sensitivities of 5–20 mm/s airflow are slightly increasing with the airflow velocity, and lower than that of airflow velocity above 20 mm/s. This means the heat transfer coefficient *h*_*v*_ does not strictly follow the empirical formula (3) at ultra-low airflow. In addition, the original data of 5–10 mm/s velocity (T_μ-TED_ = 50 °C) is displayed in Fig. 4f. The RMS of the noise is about 7.15 μV. The signal of 5 mm/s is 8 times higher than the noise level. The voltage signals of 5, 7.5, and 10 mm/s are distinguishable from each other. These results indicate the μ-TED owns an airflow detection limit of 5 mm/s, as well as a resolution of 2.5 mm/s. The numerical simulation show the μ-TED owns the potential to detect the airflow velovity of 4.03 mm/s, as shown as Supplementary Figs. S6a–S6c (Supplementary information). Besides, the stability of the airflow test is performed, as shown in Supplementary Fig. S7 (Supplementary information). The μ-TED shows good stability in the long-period test and cycle test at a device temperature of 50 °C. Notably, the as-reported μ-TED achieves a sensitivity close to reference^21^ with a sensitive area of 1 mm^2^, only 1/400 of the latter (4 cm^2^; sensitivity of 25–50 mV/(m/s) at T_μ-TED_ = 50 °C). The area of the μ-TED is significantly miniaturized, which makes the device’s temperature free of the disturbance of the external environment. Hence, the as-reported μ-TED exhibits an ultra-low airflow detection limit of 5 mm/s.

Further, the transient response of the μ-TED is studied, as shown in Fig. 4g. With the increase of the airflow rate, the voltage signal reaches the maximum value more quickly. This is because of the increase of the heat transfer coefficient *h*, following formula (3). The heat exchange is more intense, prompting the μ-TED to reach an equilibrium state faster. Figure 4h shows the response time of the μ-TED under different airflow velocities. Here, the response time is defined as the duration of the voltage signal rising from 10% to 90% of the maximum signal. The response time decreases sharply at first and finally approaches a stable value. The response time of 100 ms is achieved when the airflow velocity is higher than 140 mm/s. Furthermore, the response performance of the μ-TED working on different device temperatures is stable, which is shown in Supplementary Fig. S6e, Supplementary information. These results indicate the μ-TED exhibits a fast response in low-velocity airflow sensing.

**Fig. 5:**
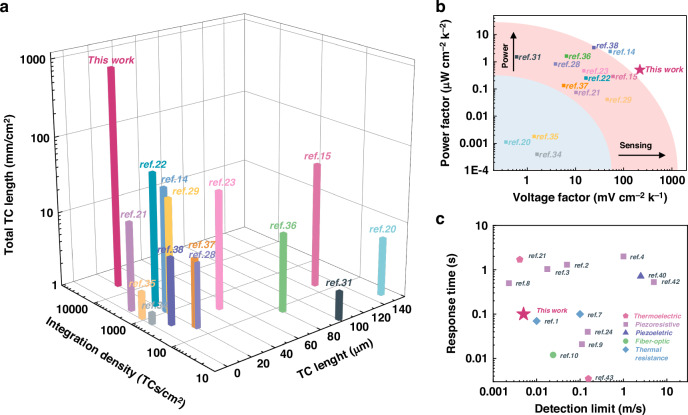
Performance comparison with recently μ-TED. The structural parameter (**a**) and thermoelectric performance (**b**) comparison of recently reported μ-TED^20,23,27–39^. **c** The airflow sensing performance of recently reported airflow sensors^1–10,24,25,40–42^

Then, the structural parameter and thermoelectric performance comparison of recently reported μ-TED is performed to illustrate the superiority of the as-reported μ-TED, as shown in Fig. 5^20,23,27–39^. Detailed data is listed in Supplementary Note S3, Supplementary information. The integration density and thermocouple’s length (namely the TE material’s thicknesses) are key structural parameters, which deeply influence the thermoelectric performance of the μ-TEDs. However, due to technological limitations, μ-TEDs of large TE thickness (>20 μm) usually result in poor integration density^15,20,23,31,36^. Here, we chose a TE thickness of around 20 μm and achieved a great TC density of 19,900 TC/cm^2^ by an elaborately designed MEMS-based process. As a result, the 2nH of as-reported μ-TED is over 10 times higher than recently reported μ-TED^20,23,27–39^. Then, Fig. 5b shows the power factor and the thermoelectric voltage factor of these μ-TEDs, which represent the power-generating ability and sensing ability, respectively. Recent works of μ-TED take much effort to improve the power-generating ability^14,15,21–23,36–38^, including thermal structure design, reducing contact resistance, and so on. However, these do not significantly influence the sensing sensitivity per unit area. Hence, the sensing sensitivity is not efficient in meeting the requirement of miniaturized applications, where the device area is limited. Our work promotes the sensing performance by increasing the effective TC length 2nH. This not only greatly improves the sensing sensitivity but is also beneficial to a good power-generating ability. As a result, the as-reported μ-TED achieves a remarkably superior thermoelectric performance of 212 mV/(K·cm^2^). At the same time, it owns a competitive power factor of 0.51 μW/(K^2^·cm^2^), compared with reference^23,28^, and^36^. These great merits provide promising potential in mini-sized systems, including micro sensors and micro energy harvest devices.

Furtherly, the airflow sensing performance is compared with recently reported airflow sensors^1–10,24,25,40–42^, as shown in Fig. 5c. Detailed data is listed in Supplementary Note S4, Supplementary information. This work is of good superiority in detection limit, as it can detect a minimum airflow of 5 mm/s, close to the ultra-sensitive graphene-based airflow sensor^8^. The response time is around 100 ms, almost 5 times faster than graphene-based piezoresistive airflow sensors. These results reveal the promising potentials of the as-reported ultra-sensitive μ-TED in quick detections of ultra-low airflow.

### Breath detection of the ultra-sensitive μ-TED-based flexible sensor

**Fig. 6:**
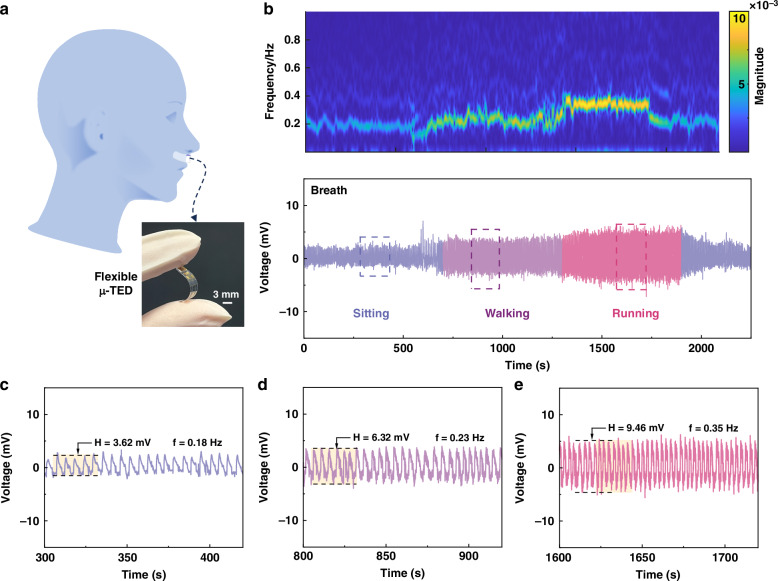
Breath detection of the μ-TED-based flexible sensor. **a** The schematic diagram of the breath detection of the μ-TED-based flexible sensor. **b** The original breath signal (bottom) and the wavelet analysis result (upper) of the μ-TED under different human motion states. **c**–**e** The enlarged picture of the sitting (blue line), walking (purple line), and running (red line) motion state marked in (**b**), respectively

The ultra-sensitive μ-TED owns an excellent heat sensing performance (Fig. 5b) even in a 1 mm^2^ sensitive area. Therefore, it is expected to achieve flexible applications due to its very small size. Figure 6a shows the schematic diagram of the breath detection with the μ-TED-based flexible sensor. Here, the flexible airflow sensor is made by directly bonding the μ-TED with a flexible PEN film, which has prepared the connecting electrode beforehand. The flexible airflow sensor is pasted under the nose to monitor the breath signal. Here, the exhaled and inhaled air can be regarded as body temperature and room temperature, respectively. They alternately flow past the μ-TED surface generating the thermoelectric signal.

The human breath detection of the μ-TED sensor is shown in Fig. 6b, containing sitting, walking, and running states. The bottom and the upper picture represent the original signal and the wavelet analysis result, respectively. The original voltage signal changes with the human state. With the increase of the exercise intensity, the breath becomes quicker and stronger. After wavelet analysis, it is found that the frequency and the magnitude show distinct differences. That means the behavior recognition is expected to be achieved with the μ-TED sensor. In addition, part signals of each motion state are extracted in Fig. 6c–e. Detailed voltage signals of exhaling and inhaling can be found in Supplementary Fig. S8a, Supplementary Information. The breathing frequency during the sitting, walking, and running motion states are 0.18 Hz, 0.23 Hz, and 0.35 Hz, respectively. And the voltage magnitudes are 3.62, 6.32, and 9.46 mV, respectively. These are in agreement with the wavelet analysis result. The ultra-sensitive μ-TED shows a promising potential for health monitoring.

The above results provide a simple demonstration of breath detection. For actual operating conditions, many specific issues should be taken into consideration. The bonding part and electrical connections between the μ-TED and flexible part should be carefully concerned, which determines the reliability and durability. Besides, the device may usually work in an environment of high humidity, even in direct contact with the sweat of the human skin. Effective encapsulation is essential to avoiding short-circuit and erosion of thermoelectric materials. Here, the Supplementary Fig. S8b–e (Supplementary Information) discussed a PDMS (Polydimethylsiloxane) encapsulated μ-TED. The device shows good water resistance (humidity). However, the sensitivity to airflow reduced a lot, which is caused by the additional thermal resistance of thick PDMS. The conformal thin film would be superior, such as parylene encapsulation. Further effort is needed to explore the potential of the μ-TED in wearable devices, including thin film encapsulation, flexible electrical connection, and so on.

## Conclusions

In summary, an ultra-sensitive μ-TED is successfully fabricated by the MEMS-based technology. High-performance Bi_2_Te_3_/Sb_2_Te_3_ TCs with a compact feature size of 25 μm are integrated by electrochemical deposition (ECD). And the TCs integration density of the μ-TED is up to 19,900 TCs/cm^2^. Benefitting from that, the μ-TED shows an excellent thermoelectric performance of 212 mV/(K·cm^2^) and a competitive power factor of 0.51 μW/(K^2^·cm^2^). Moreover, the effective total TC length 2nH is high to 796 mm/cm^2^, greatly promoting airflow sensitivity. The μ-TED exhibits an ultra-low airflow detection limit of 5 mm/s and a fast response time of 100 ms only with a 1 mm^2^ sensitive area, revealing great potential in fast detection of ultra-low airflow. Moreover, the ultra-sensitive μ-TED, due to its compact size, was employed as a flexible breath sensor. Breath signals of various human states are successfully detected, demonstrating the potential for miniaturized and portable applications. Briefly, this work achieves an ultra-sensitive μ-TED airflow sensor and demonstrates a miniaturized wearable application. For future consideration, it also reveals some issues that need to be considered. Especially, the purity of thermoelectric materials deposited in micro hole should be carefully optimized, which would provide a further improvement in thermoelectric performance. To reduce the fabrication cost, seed layer material with good acid resistance and ductility needs exploring to replace Au. Additional attempts are also required in developing the integration and encapsulation method in the wearable applications. Along with these efforts, μ-TED will provide a promising application potential in self-powered, multi-functional, and flexible electronic systems.

## Experimental section

### Fabrication details of the ultra-sensitive μ-TED

The thick photoresist used in the deposition of TE materials is AZ 4903 (high viscosity). It was spin-coated with a rotation rate of 1100 rpm. Then, it was backed at 95 °C for 40 min for curing. The thin photoresist used in the top connections is AZ 4330 (low viscosity). It was spin-coated with a rotation rate of 1600 rpm and backed at 95 °C for 10 min. The photoresist AZ 4903 and AZ 4330 are both developed with the same solution (AZ400K: H_2_O = 3:1, in volume). The Cr/Au seed layers are sputtering at a pressure of 5*10^−4 ^Pa and a power of 150 W. The seed layers of bottom and top connections are 800 nm and 300 nm, respectively. The Cr and Au layers are etched by I/KI solution and KMnO_4_ solution, respectively. The polish of thermoelectric materials is manually operated with 3 M sandpaper of 5000#.

The thermoelectric materials Bi_2_Te_3_ and Sb_2_Te_3_ are electrochemically deposited with a nitric acid-based solution^43–45^ (see Supplementary Note S2, Supplementary Information). And, they are deposited with pulsed voltage for better uniformity. The E_on_/E_off_ of Bi_2_Te_3_ and Sb_2_Te_3_ are −0.03/0.1 V and −0.195 /0.1 V, respectively. Here, the deposition potential E_on_ is examined with cyclic voltammetry (CV) curves, (see Supplementary Fig. S3a, d, Supplementary Information). And the T_on_/T_off_ of Bi_2_Te_3_ and Sb_2_Te_3_ are 0.1/0.2 s and 0.1/0.4 s, respectively.

### Numerical analysis of the airflow sensing performance

The numerical analysis of the airflow sensing process is performed with COMSOL Multiphysics. Here, the μ-TED is simplified to a single TE unit, because its TE units are under similar conditions. The TE unit parameters are shown in Supplementary Note S1 and Supplementary Fig. S1a, Supplementary information. The room temperature is set as 20 °C, and the bottom temperature of the μ-TED device is fixed at a constant temperature of 30 °C. The boundary condition of the top connection is set as convective heat flux. The heat transfer coefficient *h*_*v*_ is defined in terms of a+b*v* (*v* stands for the flow velocity)^26^. Here, the values of a and b are set as 5.7 and 3.8, the same as the ref.^26^. Supplementary Fig. S1b and S1c (Supplementary information) show the temperature and electric potential distribution of the numerical results.

### Electrical measurement methods

The airflow performance is performed with a homemade micro-syringe. The injection rate is controlled with a stepper motor. The relationship between the injection flow rate Q (μL/s) and airflow velocity *v* (mm/s) is listed in Supplementary Table S1.2 of Supplementary Note S1, Supplementary information. The thermoelectric performance is tested with a homemade temperature control platform, see Supplementary Figure S5a, Supplementary information. The electrical output is measured with a Keysight 34420A digital volt meter. Transient response of the μ-TED is detected with National Instruments 6289.

## Supplementary information

Supplemantal information
