# Supplementary material for: Ultra-sensitive micro thermoelectric device for energy harvesting and ultra-low airflow detection
Source: Microsyst Nanoeng. 2025 Apr 28;11:73. doi: 10.1038/s41378-025-00921-7 (PMC12034818; doi:10.1038/s41378-025-00921-7)
Supplement: Supplementary file 1 — Supplemantal information [file 41378_2025_921_MOESM1_ESM.docx]

**Supplementary Information**

Ultra-sensitive micro thermoelectric device for energy harvesting and ultra-low airflow detection

Bo Yan ^1,2^, Jiaxiang Wang ^1,2^, Yi Chen ^1,2^, Yahui Li ^3^, Xiangxiang Gao ^1,2^, Zhiyuan Hu ^1,2^, Xiaowen Zhou ^1,2^, Mengqiu Li ^1,2^, Zhuoqing Yang ^1^, Congchun Zhang ^1,^ ^[[1]](#footnote-1)^*

^1^ National Key Laboratory of Advanced Micro and Nano Manufacture Technology, Shanghai Jiao Tong University, Shanghai, 200240, China

^2^ Department of Micro/Nano Electronics, School of Electronic Information and Electrical Engineering, Shanghai Jiao Tong University, Shanghai, 200240, China

^3^ School of Electrical and Electronic Engineering, Nanyang Technological University, Singapore 639798, Singapore

**Content of the Supplementary information：**

Note S1: The detailed information in the airflow sensing numerical analysis and practical measurement.

Note S2: Preparations of the electrochemical solutions of Bi_2_Te_3_ and Sb_2_Te_3_.

Note S3: The detailed information of the recently reported vertical μ-TED.

Note S4: The detailed information of recently reported airflow sensors.

Figure S1. The airflow numerical analysis of the μ-TED.

Figure S2. The optical image of the compact μ-TED during each process.

Figure S3. Detailed information of electrochemical deposited Bi_2_Te_3_ and Sb_2_Te_3_.

Figure S4. Element analysis of the μ-TED.

Figure S5. Detailed information of the thermoelectric performance measurement.

Figure S6. Additional information of airflow sensing performance.

Figure S7. The stability of the μ-TED in airflow sensing application.

Figure S8. Additional Information of the breath detection application.

# Notes:

Note S1: The detailed information in the airflow sensing numerical analysis and practical measurement.

In the airflow sensing numerical analysis, the TE units of the μ-TED are in equivalent conditions under the airflow. Here, the μ-TED is simplified with a single TE unit. The thermoelectric units are built with an equal value of TE diameter and space, as shown in Figure S1. The details structural parameters of numerical analysis are shown in Table S1.1. Among that, the thickness H of the thermoelectric materials ranges from 5 to 20 μm.

Table S1.1 The structural parameters of airflow sensing numerical analysis.

| Diameter  (μm) | Spacing  (μm) | Thickness  (μm) | Integration density  (TCs/cm^2^) | 2nH  (μm/cm^2^) |
| --- | --- | --- | --- | --- |
| 200 | 200 | H | 313 | 616*H |
| 100 | 100 | H | 1250 | 2500*H |
| 75 | 75 | H | 2222 | 4444*H |
| 50 | 50 | H | 5000 | 1000*H |
| 25 | 25 | H | 20000 | 40000*H |
| H = 5, 10, 15, 20 μm | | | | |

In the practical measurement of airflow sensing performance, the ultra-low airflow is generated with a homemade micro-syringe. The micro-syringe is controlled by a programmable stepping motor. During the measurement, the air is injected into a pipe on a constant flux of Q μL/s. The μ-TED is placed at the outlet of the pipe. And inner diameter d of the outlet is 4.5 mm. The flow rate *v* mm/s at the outlet is calculated with the next formula:

$$v= \frac{Q}{(\pi d^{2}/4)}$$

The detailed relationship between the flux Q μL/s and flow rate *v* mm/s is listed in Table S1.2.

Table S1.2 Relationship of airflow velocity *v* and injected rate Q.

| Injecting rate μL/s | Outlet diameter mm | Flow velocity mm/s |
| --- | --- | --- |
| 80 | 4.5 | 5.01 |
| 119 | 4.5 | 7.51 |
| 165 | 4.5 | 10.36 |
| 199 | 4.5 | 12.52 |
| 239 | 4.5 | 15.02 |
| 280 | 4.5 | 17.61 |
| 329 | 4.5 | 20.72 |
| 631 | 4.5 | 39.71 |
| 961 | 4.5 | 60.43 |
| 1262 | 4.5 | 79.42 |
| 1592 | 4.5 | 100.13 |
| 1921 | 4.5 | 120.85 |
| 2223 | 4.5 | 139.84 |
| 2552 | 4.5 | 160.56 |

Note S2: Preparations of the electrochemical solutions of Bi_2_Te_3_ and Sb_2_Te_3_.

The electrochemical solutions of the two kinds of thermoelectric materials are prepared in Table S2. Here, nitric acid and tartaric acid are introduced to dissolve the chemicals [43, 44].

The preparation of the Bi_2_Te_3_ solution follows the next steps. Firstly, the HNO_3_ solution of 1.2 M/L is prepared. Next, the TeO_2_ is dissolved in the former solution at 60 ℃. After the solution cools down to room temperature, Bi(NO_3_)_3_·5H_2_O is added and dissolved forming the Bi_2_Te_3_ solution.

The preparation of the Sb_2_Te_3_ solution follows the next steps. Firstly, the tartaric acid solution and HNO_3_ solution are prepared. Then, the Sb_2_O_3_ and TeO_2_ are dissolved in former solutions at 60℃, respectively. After cooling down to room temperature, the former solutions are mixed and stirred into a transparent Sb_2_Te_3_ solution.

Table S2. Component composition of the electrochemical solutions.

| Chemicals | Bi_2_Te_3_ | Sb_2_Te_3_ |
| --- | --- | --- |
| Bi(NO_3_)_3_·5H_2_O | 10 mM/L | / |
| Sb_2_O_3_ | / | 10 mM/L |
| TeO_2_ | 12 mM/L | 12 mM/L |
| HNO_3_ | 1.2 M/L | 1 M/L |
| Tartaric acid | / | 0.3 M/L |

Note S3: The detailed information of the recently reported vertical μ-TED.

Table S3 provides a comprehensive comparison of recent research on vertical μ-TED, including their structural parameter, thermoelectric performance, and power generation capability. The high-integration μ-TED of this work exhibits excellent thermoelectric sensitivity (namely voltage factor).

Table S3. Summary of previously reported vertical μ-TEDs.

| Ref. | TE materials | Size  (cm^2^) | TCs | Density (TCs/cm^2^) | TE thickness (μm) | Voltage factor  (mV K^-1^cm^-2^) | Power factor (μW cm^-2^ K^-2^) |
| --- | --- | --- | --- | --- | --- | --- | --- |
| This work | Bi_2_Te_3_/Sb_2_Te_3_ | 0.01 | 199 | 19900 | 20 | 216.67 | 0.5059 |
| ref.14 | (Bi,Sb)_2_Te_3_ | 0.01 | 12 | 1071 | 20 | 52.29 | 2.3929 |
| ref.15 | Bi_2_Te_3_-based | 0.56 | 99 | 176 | 126 | 59.84 | 0.2884 |
| ref.20 | CNT-polystyrene | 100 | 1985 | 20 | 150 | 0.36 | 0.0011 |
| ref.21 | Bi_2_Te_3_/Sb_2_Te_3_ | 4 | 10082 | 2521 | 3 | 10.24 | 0.0747 |
| ref.22 | Bi_2_Te_3_/Sb_2_Te_3_ | 0.14 | 282 | 1945 | 20 | 16.44 | 0.2533 |
| ref.23 | Bi_2_Te_3_/Sb_2_Te_3_ | 1 | 364 | 364 | 50 | 14.92 | 0.4679 |
| ref.28 | Cu/Ni | 0.49 | 90 | 183 | 20 | 3.84 | 0.833 |
| ref.29 | Bi_2_Te_3_/Sb_2_Te_3_ | 0.29 | 242 | 834 | 20 | 45.46 | 0.0414 |
| ref.31 | Bi_2_Te_3_/Sb_2_Te_3_ | 3 | 8 | 2.67 | 100 | 0.6 | 1.52 |
| ref.34 | SiNW | 0.25 | 162 | 648 | 1.1 | 1.59 | 0.0004 |
| ref.35 | Bi_2_Te_3_/Sb_2_Te_3_ | 0.11 | 127 | 1155 | 1 | 1.38 | 0.0018 |
| ref.36 | Bi_2_Te_3_/Sb_x_Te_y_ | 1 | 71 | 71 | 80 | 5.58 | 1.63 |
| ref.37 | Bi_2_Te_3_/Sb_2_Te_3_ | 1 | 200 | 200 | 20 | 5.68 | 0.1343 |
| ref.38 | Bi_2_Te_3_/Sb_2_Te_3_ | 0.33 | 127 | 391 | 10 | 24 | 3.33 |

Note S4: The detailed information of recently reported airflow sensors.

Table S4 compares the sensing performance of airflow sensors reported in recent years. This work shows an ultra-low detection limit and competitive response time.

Table S4. Performance comparison of recent airflow sensors.

| Ref. | Mechanism | Detection limit (m/s) | Response time (ms) |
| --- | --- | --- | --- |
| This work | Thermoelectric | 0.005 | 100 |
| ref.21 | Thermoelectric | 0.02 | 1700 |
| ref.43 | Thermoelectric | 0.156 | 3.5 |
| ref.10 | Fiber-optic | 0.024 | 12 |
| ref.1 | Thermal resistance | 0.01 | 70 |
| ref.7 | Thermal resistance | 0.1 | 100 |
| ref.25 | Thermal resistance | 0.5 | 0.68 |
| ref.40 | Piezoelectric | 2.5 | 714 |
| ref.2 | Piezoresistive | 0.05 | 1300 |
| ref.3 | Piezoresistive | 0.0176 | 1040 |
| ref.4 | Piezoresistive | 1 | 2000 |
| ref.8 | Piezoresistive | 0.0023 | 500 |
| ref.9 | Piezoresistive | 0.11 | 21 |
| ref.42 | Piezoresistive | 5 | 530 |
| ref.24 | Piezoresistive | 0.15 | 40 |

**Figures:**


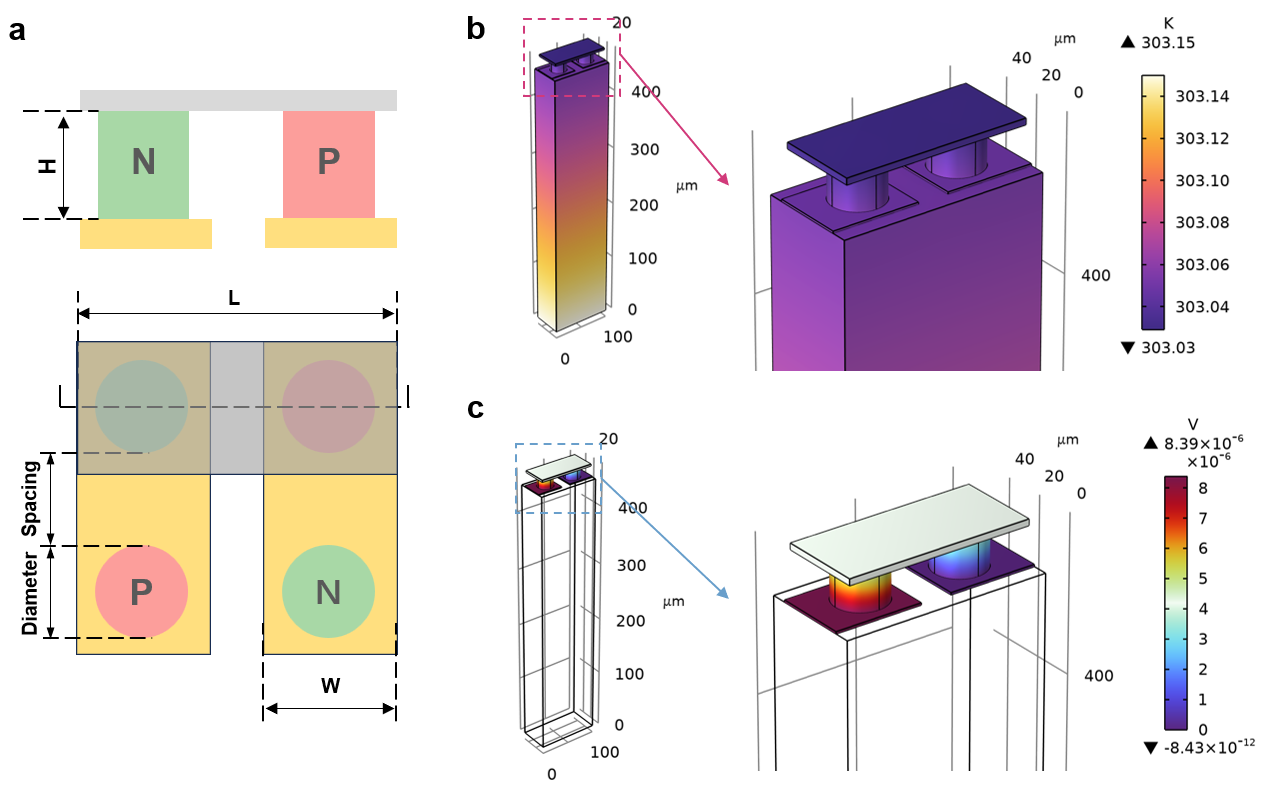


**Figure S1.** The airflow numerical analysis of the μ-TED. (a) The schematic diagram of the TE mold used in numerical analysis. The spacing between the TE element is equal to the diameter D. The width and length of the connections are D+15 μm and 3*D+15 μm, respectively. The temperature distribution (b) and electrical potential distribution (c) of the TE unit under airflow numerical analysis. The bottom temperature of the substrate is fixed at 30 ℃.


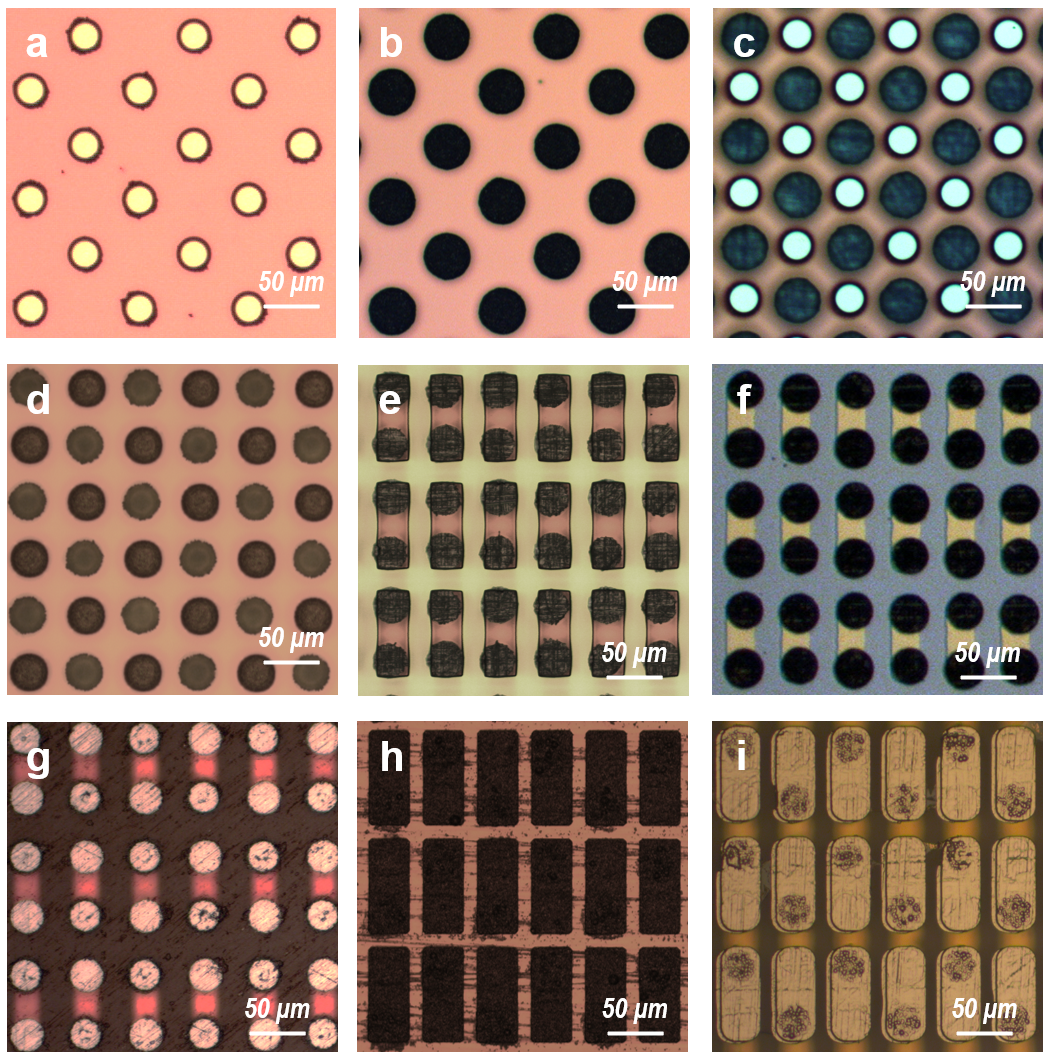


**Figure S2.** The optical image of the compact μ-TED during each process. (a) Thick photoresist with N-type pattern after photolithography. (b) Electrochemical deposited N-type Bi_2_Te_3_. (c) Photoresist with N-type pattern after photolithography. Here, N-type Bi_2_Te_3_ is covered with a thin photoresist. (d) Image after electrochemical deposited P-type Sb_2_Te_3_. (e) The residual photoresist with bottom connection patterns after photolithography. (f) Bottom connections after etched. (g) The optical image after filling thick photoresist and polishing. (h) The electroplated Ni top connections. (i) Top connection after etching and photoresist release.


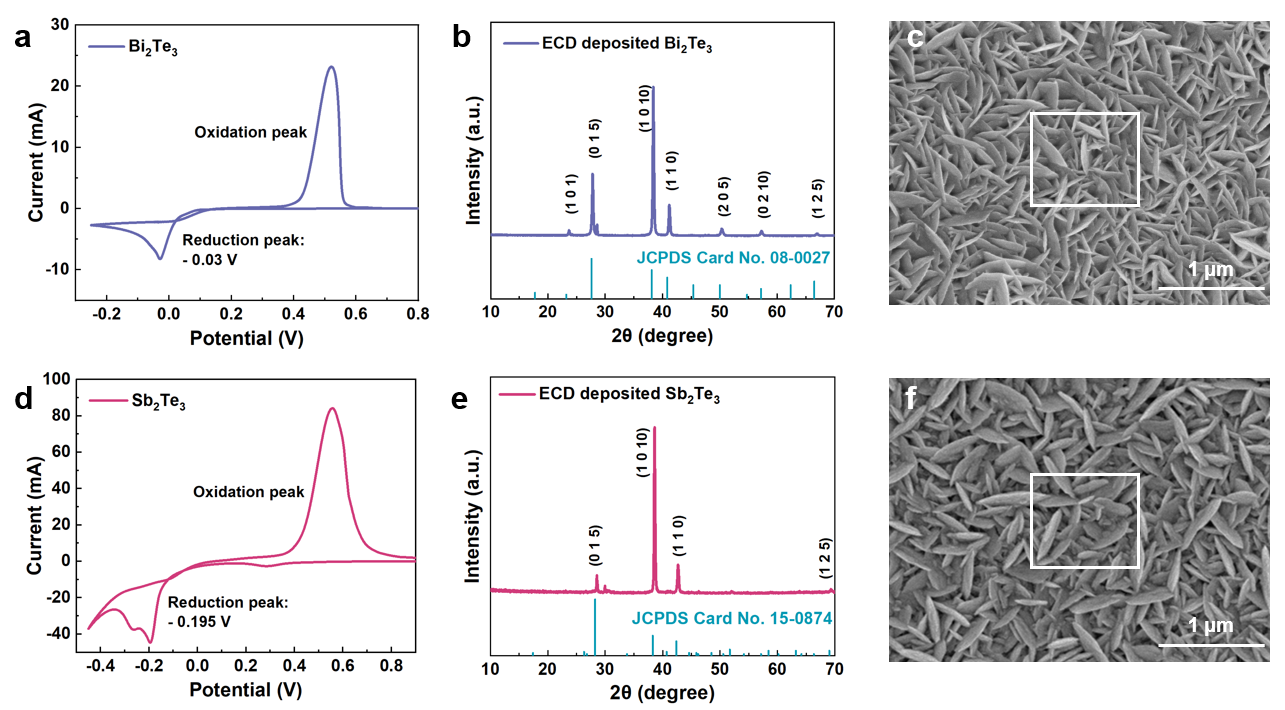


**Figure S3.** Detailed information of electrochemical deposited Bi_2_Te_3_ and Sb_2_Te_3_. (a) and (d) are the cyclic voltammetry curves of Bi_2_Te_3_ and Sb_2_Te_3_ solutions, respectively. The reduction peak of Bi_2_Te_3_ is around -0.03 V, and the reduction of Sb_2_Te_3_ occurs at -0.195 V. They are the corresponding deposition potentials E_on_ of Bi_2_Te_3_ and Sb_2_Te_3_, respectively. (b) and (e) are the XRD patterns of Bi_2_Te_3_ and Sb_2_Te_3_ samples, respectively. Here, the samples are ECD deposited on a flat surface without micro-structure. The characteristic peaks of the Bi_2_Te_3_ and Sb_2_Te_3_ samples are well-matched with the JCPDS card. It indicates the thermoelectric materials Bi_2_Te_3_ and Sb_2_Te_3_ are successfully fabricated. (c) and (f) are the SEM images of Bi_2_Te_3_ and Sb_2_Te_3_ samples. They're both needle-like structures. Besides, the EDS is performed three times at different areas of each sample. The average Bi:Te ratio of Bi_2_Te_3_ sample is determined to be 2:3.12. The average Sb:Te ratio of Sb_2_Te_3_ sample is determined to be2:3.15. These results indicate the thermoelectric materials are successfully prepared.


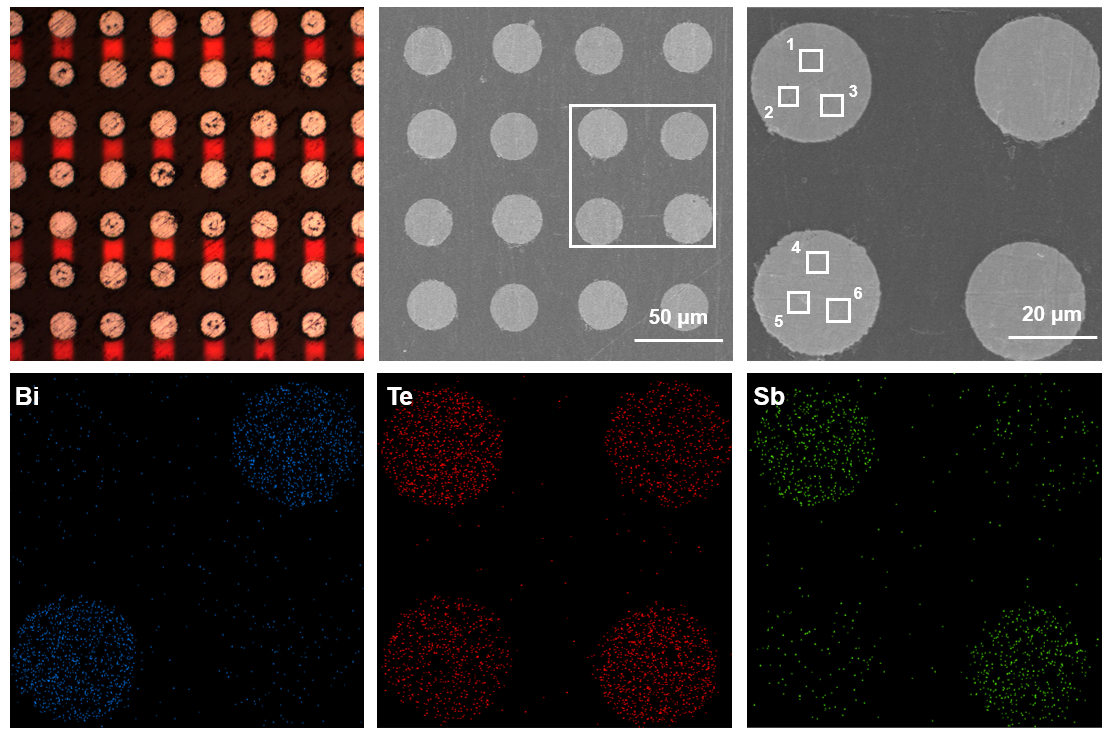


**Figure S4.** Elemental analysis of the μ-TED. Here, the μ-TED filled with photoresist is polished exposing the surface of P and N thermoelectric (TE) structures. And energy dispersive spectrometer (EDS) test is performed. The element mapping of the Bi, Te, and Sb indicates the TE structures are fabricated in the right area. Additionally, spot scanning of TE structures is performed to investigate the element composition. The result is shown in Table S6. The element ratio of N-type material is determined to be Bi:Te = 2:2.93, indicating that Bi_2_Te_3_ is successfully integrated. But, the P-type material is determined to be Sb:Te = 2:4.56. There is no other possible chemical compound composed of Sb and Te. Hence, the P-type TE material may be composed of 76% Sb_2_Te_3_ and 24% Te. This is not consistent with the flat deposited samples (Figure S3). We think it is due to the slow diffusion velocity of Sb^3+^, which is indirectly dissolved in the ECD solution through a tartaric (organic) acid. In the micro-size opening, the diffusion of Sb^3+^ is not efficient for the Sb_2_Te_3_ deposition. Additional adjustment is required for the ECD deposition of micro-size Sb_2_Te_3_ structure.

Table S5. Element composition of the P and N materials.

| Spot/Atom% | 1 | 2 | 3 | 4 | 5 | 6 |
| --- | --- | --- | --- | --- | --- | --- |
| Bi | 0.00 | 0.88 | 0.33 | 37.14 | 36.19 | 38.47 |
| Te | 72.05 | 69.20 | 67.03 | 59.26 | 60.17 | 58.94 |
| Sb | 27.95 | 29.92 | 32.64 | 3.61 | 3.63 | 2.59 |
| Ratio | Sb:Te = 2:4.56 | | | Bi:Te = 2:2.93 | | |


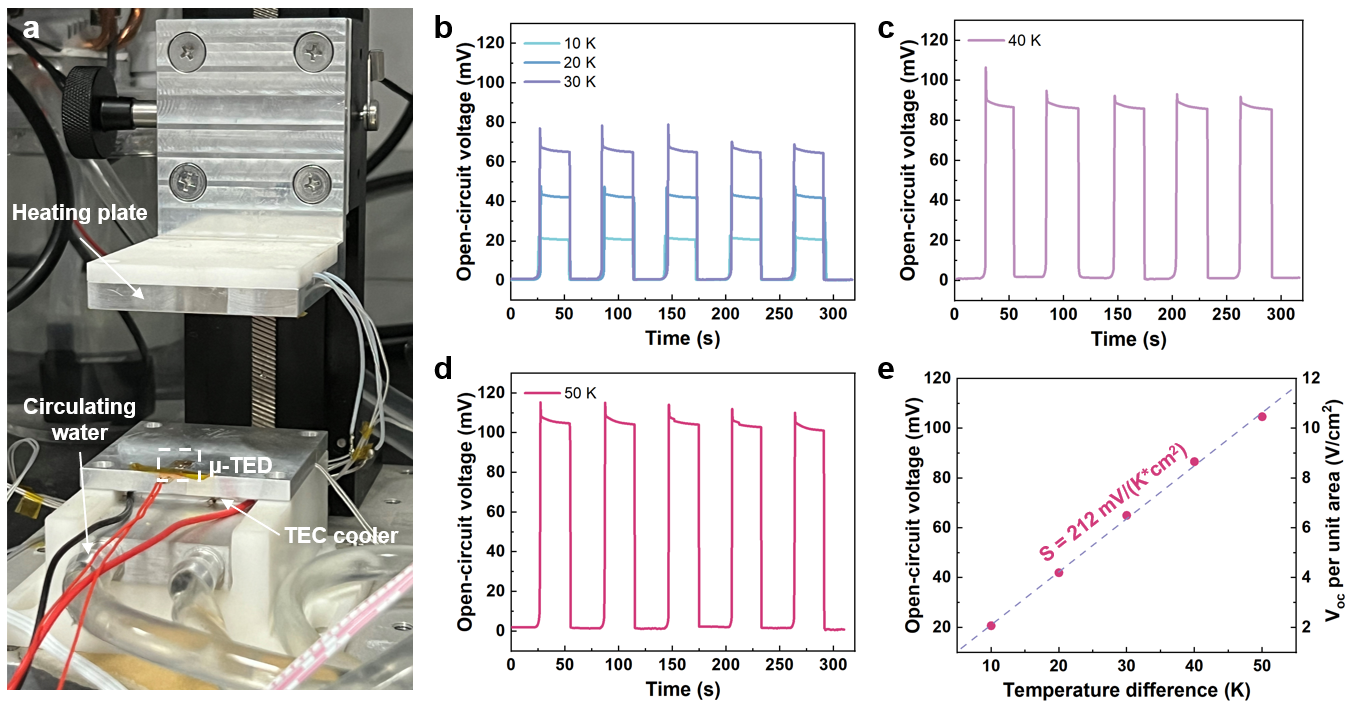


**Figure S5.** Detailed information of the thermoelectric performance measurement. (a) The homemade dual temperature control platform for loading vertical temperature difference. The upper plate is heated with a metal ceramics heater. The bottom plate is heated or cooled by a thermoelectric cooler. The waste heat is removed by circulating water. (b) - (d) The cycle tests of the μ-TED under a temperature difference of 10 - 50 K. The thermoelectric potential of each temperature difference shows good consistency. (e) The relationship between the open-circuit voltage and temperature difference. The sensitivity of the μ-TED is calculated to be 212 mV/(K*cm^2^).


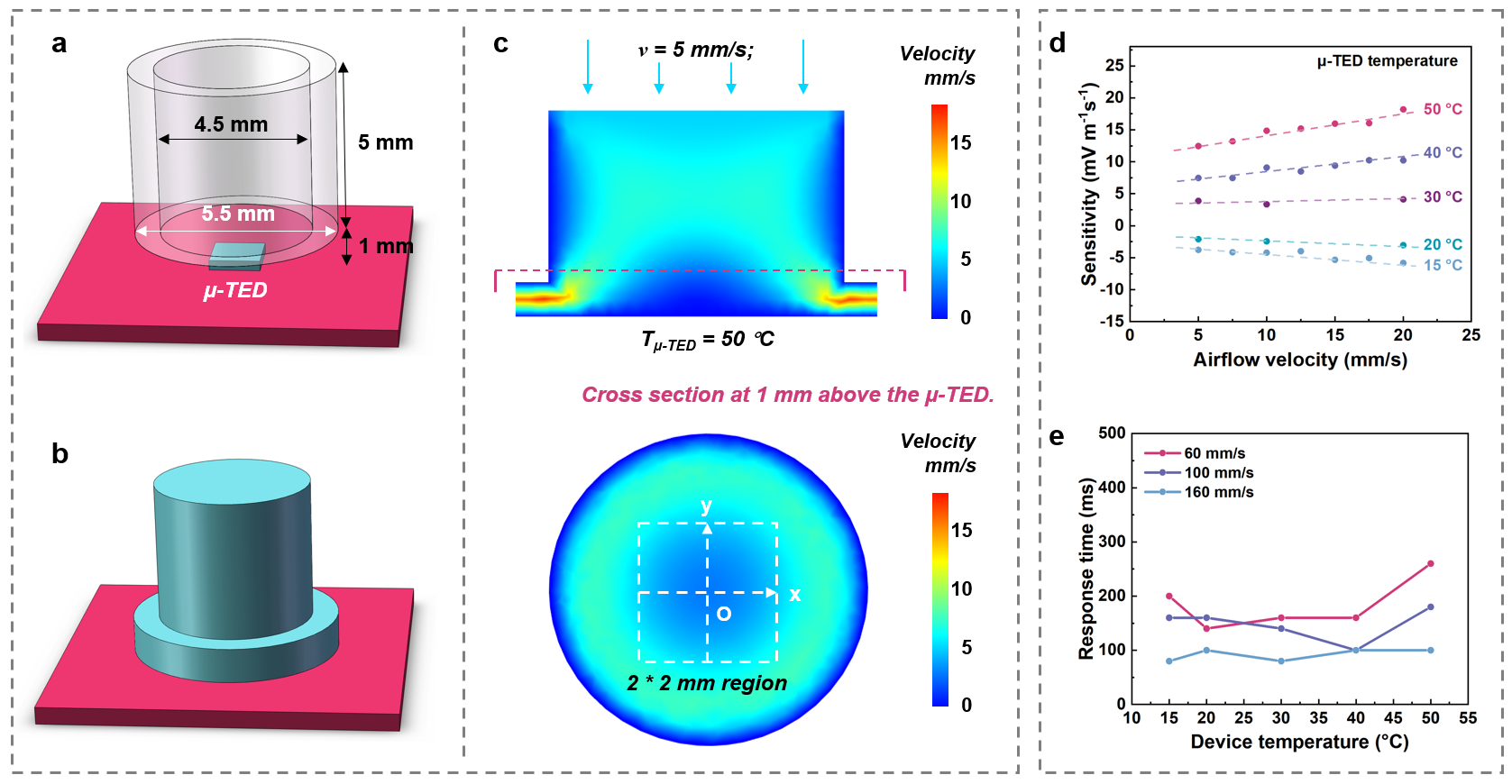


**Figure S6.** Additional information of airflow sensing performance. (a) The experiment set-up of the airflow test. The bottom of the chamber is 1 mm above the heating plate. (b) The airflow field region in the chamber. (c) The airflow velocity distribution. The top is the central plane of the chamber, the bottom is the plane 1 mm above the μ-TED substrate. (d) The sensitivities for airflow velocities of 5-20 mm/s under μ-TED temperature of 15-50 ℃. The sensitivities are slightly lower than airflow velocity above 20 mm/s. This indicates the heat transfer coefficient *h_v_* does not strictly follow the empirical formula (3) at ultra-low airflow. (e) The response time of the compact μ-TED changes with the device temperature. The response time of the 50 ℃ μ-TED shows a relatively steady response speed (around 100 ms).

Table S6. The simulation airflow velocity at different position of Figure S6b. (unit: mm/s)

| x  y | -1 | 0 | 1 |
| --- | --- | --- | --- |
| 1 | 4.41 | 3.88 | 4.43 |
| 0 | 3.87 | 3.26 | 3.87 |
| -1 | 4.41 | 3.83 | 4.32 |


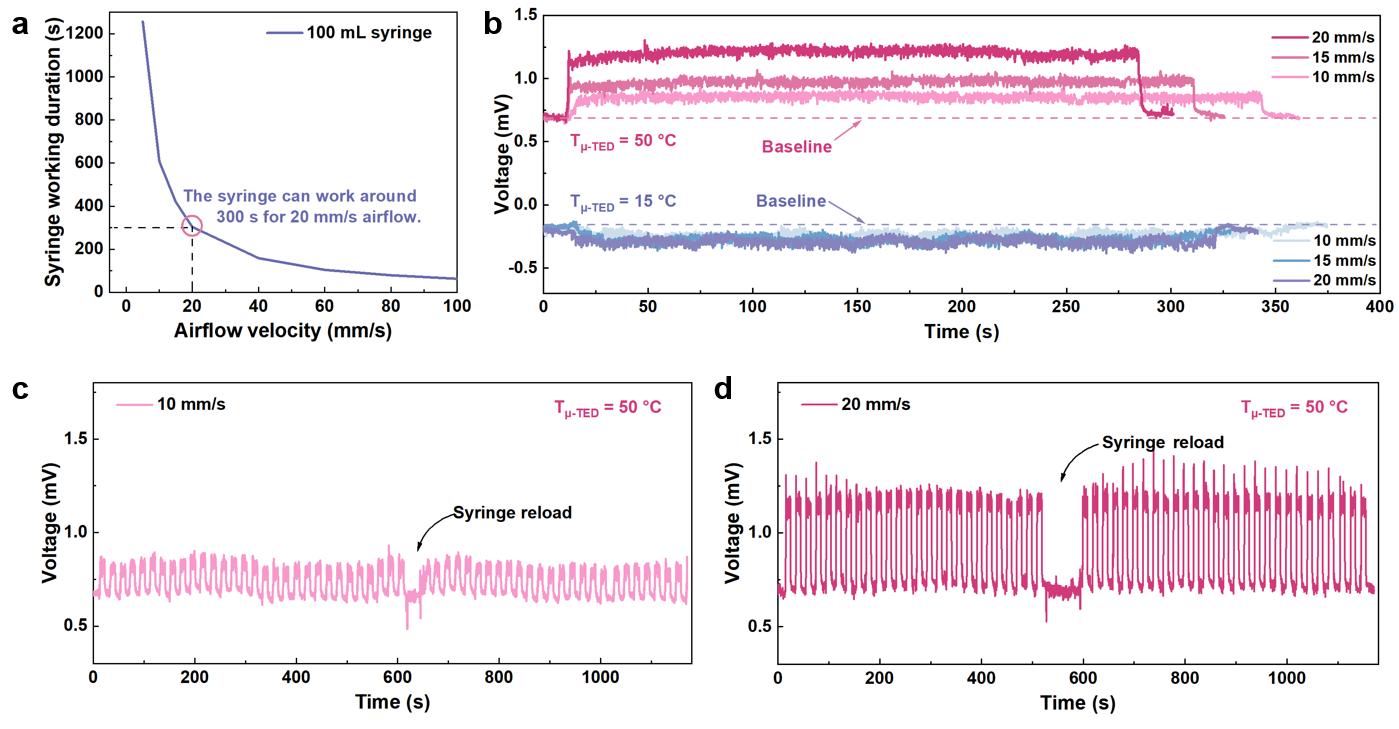


**Figure S7.** The stability of the μ-TED in airflow sensing application. (a) The relationship between the maximum syringe working duration and airflow velocity. The airflow generator has a maximum capacity of 100 ml, which limits the working duration at different airflow velocities. For a long-period test, low airflow velocity (<= 20 mm/s) is chosen. (b) The airflow test of 10 - 20 mm/s over 5 min. When T_μ-TED_ (such as 15 ℃) is close to the room temperature, the detected voltages are hard to distinguish from each other. In the term of T_μ-TED_ = 50 ℃, the detected voltages are relatively stable, and can be distinguished at airflow of 5 mm/s interval. Figure S7 (c) and (d) are the cycle tests of 10 and 20 mm/s airflow over a long time. The T_μ-TED_ is 50 ℃. The signal of 20 mm/s shows a better consistency than 10 mm/s. The long-time test is mainly influenced by the disturbance of airflow temperature (room temperature), which is not strictly controlled.


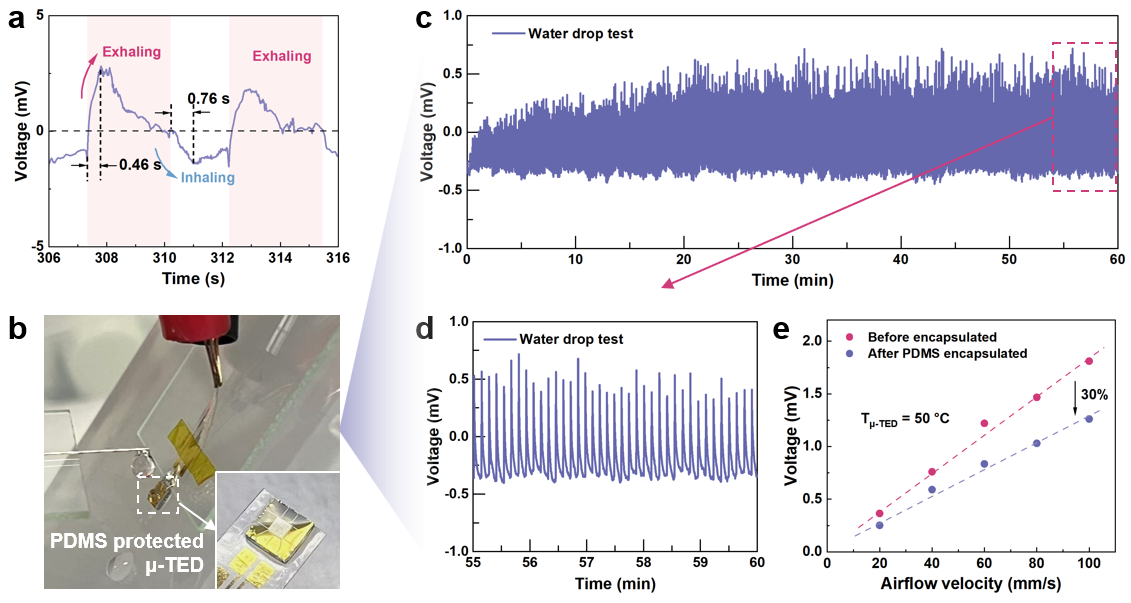


**Figure S8.** Additional Information of the breath detection application. (a) The enlarged image of the breath signal. (b) The PDMS (Polydimethylsiloxane) encapsulated μ-TED for the water test. This experiment is used to indirectly demonstrate the humidity resistance of the encapsulated μ-TED. A 10 μm PDMS (curing agent ratio: 10%) is spin-coated on the μ-TED, followed by exhausting gas in vacuum. And it is cured at 80 ℃ for 30 min. Then, the voltage signal is recorded with water constantly dripping on the μ-TED. Figure S8 (c) and (d) are the voltage signal of the water test over one hour. The water drop is well detected, indicating the PDMS encapsulated μ-TED possesses good humidity resistance. (e) The airflow test of μ-TED before and after PDMS encapsulated. The voltage is reduced by 30%, which is due to the heat loss on the thick PDMS. Thin film encapsulation may help to reduce the negative impact, such as parylene (CVD).

**References**

[1] Sadeghi M.M., Peterson R.L., and Najafi K. Air flow sensing using micro-wire-bonded hair-like hot-wire anemometry. *J. Micromech. Microeng.* **23**, 085017 (2013).

[2] Wang H., et al. Bioinspired fluffy fabric with in situ grown carbon nanotubes for ultrasensitive wearable airflow sensor. *Adv. Mater.* **32**, 1908214 (2020).

[3] Zhou W., et al. Bionic adaptive thin‐membranes sensory system based on microspring effect for high‐sensitive airflow perception and noncontact manipulation. *Adv. Funct. Mater.* **31**, 2105323 (2021).

[4] Park J., et al. Giant tunneling piezoresistance of composite elastomers with interlocked microdome arrays for ultrasensitive and multimodal electronic skins. *ACS Nano* **8**, 4689-4697 (2014).

[5] Yu Y. D., et al. Wearable respiration sensor for continuous healthcare monitoring using a micro-thermoelectric generator with rapid response time and chip-level design. Adv. Mater. Technol. 7, (2022).

[6] Jiang T., et al. Wearable breath monitoring via a hot-film/calorimetric airflow sensing system. *Biosensors & Bioelectronics* **163**, 112288 (2020).

[7] Zhao S. and Zhu R. Electronic skin with multifunction sensors based on thermosensation. *Adv. Mater.* **29**, 1606151 (2017).

[8] Huang L., et al. Ultrasensitive, fast-responsive, directional airflow sensing by bioinspired suspended graphene fibers. *Nano Lett.* **23**, 597-605 (2023). s

[9] Jiang Q., et al. Ultrasensitive airflow sensors based on suspended carbon nanotube networks. *Adv. Mater.* **34**, 2107062 (2022).

[10] Zhang Z., et al. A multifunctional airflow sensor enabled by optical micro/nanofiber. *Adv. Fiber Mater.* **3**, 359-367 (2021).

[11] Pourkiaei S.M., et al. Thermoelectric cooler and thermoelectric generator devices: A review of present and potential applications, modeling and materials. *Energy* **186**, 115849 (2019).

[12] Shi X.L., Zou J., and Chen Z.G. Advanced thermoelectric design: From materials and structures to devices. *Chem Rev* **120**, 7399-7515 (2020).

[13] Yan J., et al. Review of micro thermoelectric generator. *J. Microelectromech. Syst.* **27**, 1-18 (2018).

[14] Bottner H., et al. New thermoelectric components using microsystem technologies. *J. Microelectromech. Syst.* **13**, 414-420 (2004).

[15] Glatz W., et al. Bi_2_te_3_-based flexible micro thermoelectric generator with optimized design. *J. Microelectromech. Syst.* **18**, 763-772 (2009).

[16] Snyder G.J., et al. Thermoelectric microdevice fabricated by a mems-like electrochemical process. *Nat Mater* **2**, 528-531 (2003).

[17] Yuan J.F. and Zhu R. A fully self-powered wearable monitoring system with systematically optimized flexible thermoelectric generator. *Appl. Energy* **271**, (2020).

[18] Zhu W., Deng Y., and Cao L. Light-concentrated solar generator and sensor based on flexible thin-film thermoelectric device. *Nano Energy* **34**, 463-471 (2017).

[19] Zhang X., et al. Stamp‐like energy harvester and programmable information encrypted display based on fully printable thermoelectric devices. *Adv. Mater.* **35**, (2022).

[20] Suemori K., Hoshino S., and Kamata T. Flexible and lightweight thermoelectric generators composed of carbon nanotube–polystyrene composites printed on film substrate. *Appl. Phys. Lett.* **103**, 153902 (2013).

[21] Liu Z., et al. High-performance integrated chip-level thermoelectric device for power generation and microflow detection. *Nano Energy* **114**, 108611 (2023).

[22] Yan B., et al. Heat-sink-free solar-driven thermoelectric device for micro energy scavenging. *Nano Energy* **123**, 109382 (2024).

[23] Yu Y., et al. High-integration and high-performance micro thermoelectric generator by femtosecond laser direct writing for self-powered iot devices. *Nano Energy* **93**, 106818 (2022).

[24] Huang S., et al. Compact biomimetic hair sensors based on single silicon nanowires for ultrafast and highly-sensitive airflow detection. *Nano Lett.* **21**, 4684-4691 (2021).

[25] Talbi A., et al. A micro-scale hot wire anemometer based on low stress (Ni/w) multi-layers deposited on nano-crystalline diamond for air flow sensing. *J. Micromech. Microeng.* **25**, 125029 (2015).

[26] Sartori E. Convection coefficient equations for forced air flow over flat surfaces. *Sol. Energy* **80**, 1063-1071 (2006).

[27] Curtin B.M., Fang E.W., and Bowers J.E. Highly ordered vertical silicon nanowire array composite thin films for thermoelectric devices. *J. Electron. Mater.* **41**, 887-894 (2012).

[28] Glatz W., Muntwyler S., and Hierold C. Optimization and fabrication of thick flexible polymer based micro thermoelectric generator. *Sens. Actuators, A* **132**, 337-345 (2006).

[29] Kim M.-Y. and Oh T.-S. Thermoelectric power generation characteristics of a thin-film device consisting of electrodeposited n-Bi_2_Te_3_ and p-Sb_2_Te_3_ thin-film legs. *J. Electron. Mater.* **42**, 2752-2757 (2013).

[30] Kim S.J., et al. Post ionized defect engineering of the screen-printed Bi_2_Te_2.7_Se_0.3_ thick film for high performance flexible thermoelectric generator. *Nano Energy* **31**, 258-263 (2017).

[31] Kim S.J., We J.H., and Cho B.J. A wearable thermoelectric generator fabricated on a glass fabric. *Energy Environ. Sci.* **7**, (2014).

[32] Kim Y.J., et al. Realization of high‐performance screen‐printed flexible thermoelectric generator by improving contact characteristics. *Adv. Mater. Interfaces* **4**, (2017).

[33] Leonov V., et al. Thermoelectric converters of human warmth for self-powered wireless sensor nodes. *IEEE Sens. J.* **7**, 650-657 (2007).

[34] Li Y., et al. Improved vertical silicon nanowire based thermoelectric power generator with polyimide filling. *IEEE Electron Device Lett.* **33**, 715-717 (2012).

[35] Mu E., et al. Fabrication and characterization of ultrathin thermoelectric device for energy conversion. *J. Power Sources* **394**, 17-25 (2018).

[36] Roth R., et al. Design and characterization of micro thermoelectric cross-plane generators with electroplated bi2te3, sbxtey, and reflow soldering. *J. Microelectromech. Syst.* **23**, 961-971 (2014).

[37] Yu Y., et al. Towards high integration and power density: Zigzag-type thin-film thermoelectric generator assisted by rapid pulse laser patterning technique. *Appl. Energy* **275**, (2020).

[38] Zhang W., Yang J., and Xu D. A high power density micro-thermoelectric generator fabricated by an integrated bottom-up approach. *J. Microelectromech. Syst.* **25**, 744-749 (2016).

[39] Lee B., et al. High-performance compliant thermoelectric generators with magnetically self-assembled soft heat conductors for self-powered wearable electronics. *Nat Commun* **11**, 5948 (2020).

[40] Bian Y., Liu R., and Hui S. Fabrication of a polyvinylidene difluoride fiber with a metal core and its application as directional air flow sensor. *Funct. Mater. Lett.* **09**, 1650001 (2016).

[41] Wang Y.-H., Lee C.-Y., and Chiang C.-M. A mems-based air flow sensor with a free-standing micro-cantilever structure. *Sensors* **7**, 2389-2401 (2007).

[42] Xue D., et al. Single-side fabricated p+si/al thermopile-based gas flow sensor for ic-foundry-compatible, high-yield, and low-cost volume manufacturing. *IEEE Trans. Electron Devices* **66**, 821-824 (2019).

[43] Martı́n-González M.S., et al. Insights into the electrodeposition of Bi_2_Te_3_. *J. Electrochem. Soc.* **149**, (2002).

[44] Trung N.H., et al. Synthesis and evaluation of thick films of electrochemically deposited Bi_2_Te_3_ and Sb_2_Te_3_ thermoelectric materials. *Materials (Basel)* **10**, 154 (2017).

[45] Trung N.H., Toan N.V., and Ono T. Flexible thermoelectric power generator with y-type structure using electrochemical deposition process. Appl. Energy 210, 467-476 (2018).

1. * Corresponding author (Congchun Zhang). E-mail: zhcc@sjtu.edu.cn [↑](#footnote-ref-1)
